# Preservation of the inferior alveolar vasculonervous bundle in mandibular resective therapies: systematic review and report of two cases

**DOI:** 10.4317/medoral.26239

**Published:** 2023-10-12

**Authors:** Pedro Tapia, Gustavo Matus-Miranda, Fernanda Díaz, Pablo Arrué

**Affiliations:** 1 Department of Maxillofacial Surgery, Red Salud Vitacura Clinic, Santiago, Chile; 2 Department of Maxillofacial Surgery, Hospital Regional Libertador Bernardo O'Higgins, Rancagua, Chile; 3 ORCID: 0000-0003-0966-7576. Cathedra of Maxillofacial Surgery and Traumatology, Universidad Andres Bello, Viña del Mar, Chile; 4 Dental Surgeon, Universidad de Valparaíso, Valparaíso, Chile; 5 Department of Maxillofacial Surgery, ICROF Clinic, Santiago, Chile

## Abstract

**Background:**

Segmental surgical resection is a frequently indicated procedure to treat aggressive mandibular tumors. One of the most important complications derived from this technique is permanent paresthesia of the inferior alveolar nerve (IAN), which significantly affects the quality of life of patients who experience it. This could be avoided through maneuvers that preserve the IAN. The objective of this paper is to review the main techniques for IAN preservation and to present 2 cases with the technique used by the author.

**Material and Methods:**

A systematic review was performed according to the PRISMA guidelines, apropos of two clinical cases reported in this study. The MEDLINE/PubMed and Scopus databases were searched. Several variables were considered and are presented in detail in the form of Tables and Figures. In addition, 2 case reports with NAI preservation techniques are presented.

**Results:**

13 articles were finally obtained for analysis. 127 patients were evaluated, reporting mandibular resections associated with various pathologies. Various surgical techniques were used, all with the same goal of maintaining the IAN. In most of the patients, the maintenance of sensitivity was achieved, which was verified with different methods.

**Conclusions:**

Preservation of the IAN in maxillofacial surgical procedures where surgical resection of the mandibular bone has been performed is an alternative that has demonstrated successful results in terms of reducing postoperative sequelae and is currently positioned as a necessary and feasible procedure.

** Key words:**Mandibular nerve, mandible, maxillofacial injuries, oral pathology, oral surgery.

## Introduction

The inferior alveolar nerve (IAN) is one of the branches of the mandibular division of the trigeminal nerve (1). It runs inside the mandibular canal and innervates the mandibular teeth, chin, lower lip, mucosa, and gingiva up to the lower second premolar (2). Lesions in the IAN are a clinical problem that have been documented in surgical procedures such as extraction of the lower third molars, mandibular fractures, orthognathic surgery, placement of dental implants, and mandibular resections due to benign and malignant tumors (1,3).

Segmental surgical resection is a procedure frequently indicated for the treatment of aggressive mandibular tumors (4). One of the most important complications derived from this technique is permanent hypoesthesia of the IAN (4). This condition significantly affects the quality of life of patients who experience it, producing alterations in speech, taste, mastication, oral hygiene, and lip competence, the latter of which is involved in the retention and swallowing of food (3). In addition, injury to this nerve can cause chronic neuropathic pain (5), adverse psychological effects, and decreased quality of life in patients (2). The complications described above could be avoided through maneuvers that preserve the IAN (4). In view of the above, the aim of this paper is to present two cases describing the surgical technique of mandibular splitting, which favors the dissection and preservation of this neurovascular bundle, in addition to a review of the literature to detail its management, the different techniques, and post-surgical results.

## Material and Methods

- Systematic review

Study design

A systematic review was performed according to the Preferred Reporting Items for Systematic Reviews and Meta-Analyses (PRISMA) guidelines, apropos of two clinical cases reported in this study. The research question to be answered was: " What are the techniques applied in the literature and their success rate in the preservation of the inferior alveolar nerve in mandibular resective therapies?".

Eligibility criteria

The eligibility criteria used to select the studies were: full text, without language restriction, and reporting preservation of the IAN or the inferior alveolar neurovascular bundle in partial or total mandibular resective therapies. Prospective, comparative, and retrospective cohort studies; clinical studies (randomized or non-randomized); case series; case reports; and technical notes were included, with no time limit. The exclusion criteria were: animal studies; articles that reported IAN preservation in enucleations, orthognathic surgeries, or non-resective pathology therapies; narrative reviews; systematic reviews; and *in vitro* studies.

Sources of information

The MEDLINE/PubMed and Scopus databases were searched for potentially relevant articles. G.M.M. and F.D.S. conducted the search independently between June 24 and 26, 2023.

Search strategy

According to the protocol described, the selected databases were searched electronically with the following keywords: "Alveolar inferior nerve AND preservation" and "Alveolar inferior nerve AND preservation AND resective therapy".

Article selection

Articles were selected independently by two reviewers (G.M.M. and F.D.S.). The main data were exported to the reference manager Mendeley. The two reviewers independently analyzed the titles and abstracts and identified articles eligible for full review. Disagreements were resolved by consensus and discussion by the two reviewers together with a third reviewer (P.T.C.) who acted as a judge to settle the disagreements generated.

Data extraction

According to the collection and extraction of data from each of the included studies, several variables were considered, which were collected in Microsoft Excel and are presented in detail in the form of Tables and Figures.

Risk of Bias

Bias analysis was not performed in this study because most of the articles studied are clinical cases or case series, so a high risk of bias is assumed.

- Cases Reports

Two clinical cases were analyzed, which were operated on for resective mandibular pathologies in 2018. Patient demographics, pathology affecting the mandible, and surgical technique of inferior alveolar nerve preservation were emphasized.

Informed consents were signed for the use of patient information and photograph. The identity of the patients remained anonymous according to the ethical principles of the Declaration of Helsinki.

## Results

- Systematic Review

**Figure 1:**
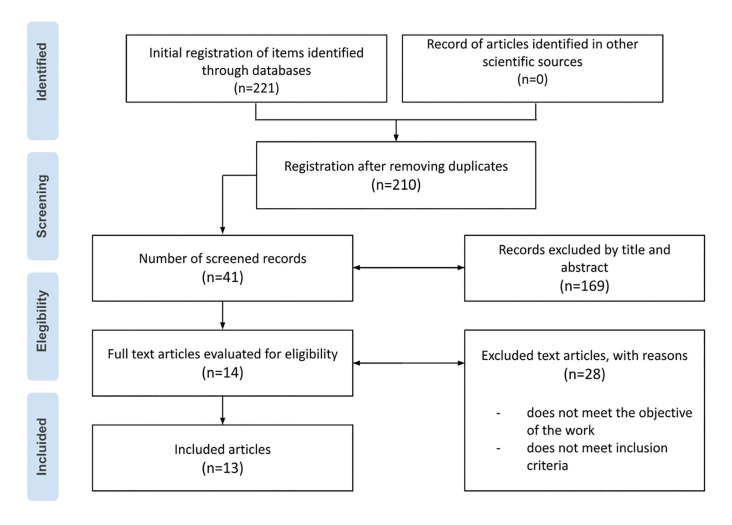
PRISMA Flow diagram.

In the initial identification process, 221 potential articles were found for review, of which 11 duplicates between the databases were eliminated. Thus, 210 publications were subjected to an in-depth review of the title and abstract, resulting in a total of 41 potential manuscripts for full-text evaluation. When applying the exclusion and inclusion criteria, 27 articles were excluded, so 13 articles were considered for analysis (Fig. 1).

Of the included articles, three are prospective cohort studies (5-7), one is a retrospective cohort study (8), one is a retrospective analysis (9), three are case series (2,10,11), four are case reports (12-15), and one is a technical note (4). The articles included a total of 127 patients, all of whom underwent mandibular resective therapies. Patient demographics are described in Table 1.

**Table 1:**
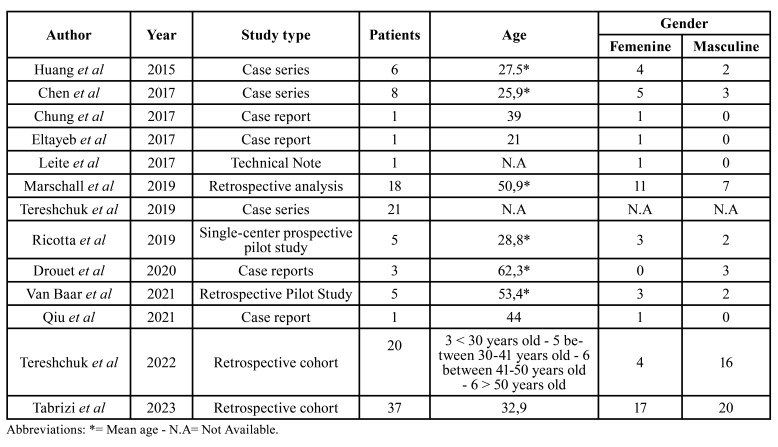
Epidemiological data of studies included in the systematic review.

Among the pathologies reported were ameloblastoma and its various types, osteoradionecrosis, ossifying fibroma, osteomyelitis, fibrous dysplasia, cemento-ossifying fibroma, drug-associated osteonecrosis, hemimandibular hyperplasia, refractory infection after osteofibrous hyperplasia, and desmoplastic fibroma (Table 2).

**Table 2:**
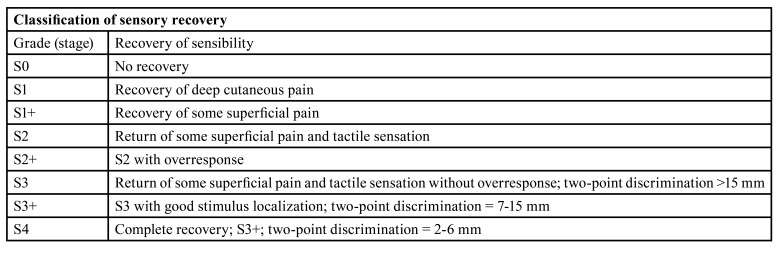
MRCS Scale to assess functional sensory recovery.

Of the 127 patients, 89 underwent immediate reconstruction, in 1 case no osteosynthesis material was used because it was a marginal partial resection of the mandible, and 37 patients were operated on with reconstruction plates only. The alveolar nerve preservation techniques are detailed in Table 3.

Regarding complications, only one laceration of the IAN was reported intraoperatively, at the time of nerve isolation. It was resolved immediately with a 9-0 nylon epineural suture. In addition, Tereshchuk *et al*, reported that in 3 patients the nerve could not be preserved (2).

Various methods were used to measure pre- and postoperative sensitivity, including a neurometer; a numerical scale based on tactile sensation and the thermalgesic scale; the Douleur Neuropathique-4 items (DN4) survey for the neuropathic component of pain; Semmes-Winstein monofilaments; the touch, direction, and two-point discrimination test; an electro-odontometer applied to the lip; and the Visual Analog Scale (VAS). The results were mostly favorable and are described in Table 3. The postoperative follow-up time ranged from 6 weeks to 45 months.

- Cases reports

**Figure 2:**
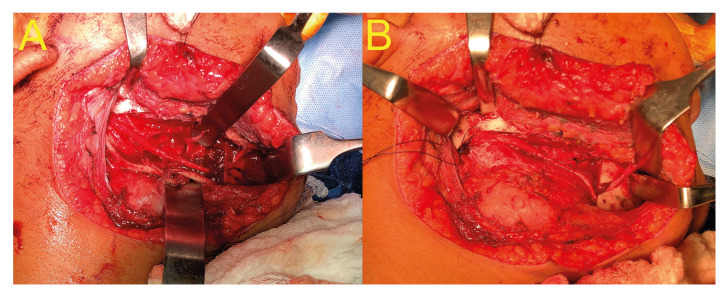
A) Resection of the vestibular cortex in pediatric follicular ameloblastoma. B) Dissection of the inferior alveolar vasculonervous bundle and resection of the mandibular inner cortex.

An 11-year-old male patient, with no general morbid history, was referred for evaluation from private practice to the institute of surgery and orofacial rehabilitation in the city of Santiago de Chile. Extraoral examination revealed asymptomatic right mandibular enlargement. The patient underwent a panoramic radiograph, which revealed an extensive multilocular radiolucent area in the mandibular body and the right mandibular ramus, with displacement of his permanent teeth. A bone biopsy was performed and the histopathological diagnosis was "pediatric follicular ameloblastoma." A block resective surgery of the body and right mandibular ramus was planned with 8 mm safety margins in an anterior-posterior direction. The procedure was performed in the central ward under general anesthesia. To preserve the alveolar nerve, a splitting technique was used, which consisted of removing the vestibular mandibular cortex with piezoelectric surgical instruments (Fig. 2), followed by dissection of the inferior alveolar neurovascular bundle and then resection of the internal cortex (Fig. 2). Subsequently, reconstruction of the mandibular defect was performed with a non-microvascularized anterior iliac crest autograft. The surgery concluded without complications. The patient was discharged 4 days after surgery. Clinical and radiographic follow-up was performed at 2, 4, 6, 12, and 24 months after surgery, during which time the sensitivity of the IAN progressively improved, reaching grade S4 on the Medical Research Council Scale (MRCS) (Table 2).

On the other side, a 54-year-old female patient with a morbid history of osteoporosis and a history of oral ibandronate consumption during 2018 was referred for evaluation from private practice in the city of Santiago de Chile, due to a cutaneous fistula in the right mandibular area. Intraoral examination showed mandibular bone sequestration, caused by osteonecrosis associated with medication. The patient underwent computed tomography (CT) of the facial massif, which showed a pathological fracture of the mandibular body on the right side with involvement of the basilar border.

**Figure 3:**
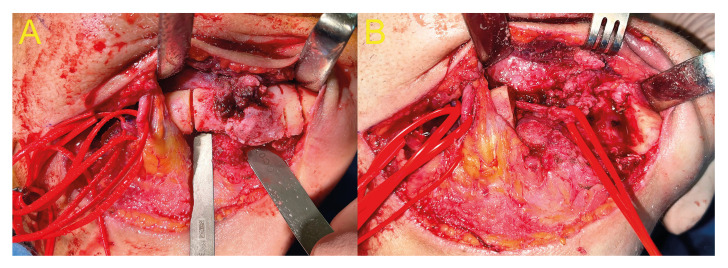
A) Peripheral vestibular, alveolar and basilar osteotomy for split vestibular cortex in osteonecrosis of the mandible. B) Dissection and preservation of inferior alveolar vasculonervous bundle after resection of necrotic mandibular segment.

The patient was hospitalized and treated with intravenous antibiotics (ampicillin/sulbactam 3 g every 6 hours) for 5 days prior to surgical treatment. Due to the impossibility of conservative treatment, surgery was planned in the central ward under general anesthesia. In the first surgical stage, the necrotic bone block was resected by splitting to preserve the IAN (Fig. 3). Subsequently, the residual defect was reconstructed with a thermocured acrylic block that allowed maintaining the volume of the tissue to be reconstructed, and a 2.4-mm-thick reconstruction plate was positioned. The patient showed good postoperative evolution, so the second surgical stage was performed 6 months later: reconstruction with an anterior iliac crest autograft. The surgery concluded without complications, and the patient was discharged 8 days after surgery. Clinical and radiographic follow-up was performed at 2, 4, 6, 12, and 24 months after surgery, during which time the IAN sensitivity progressively improved, reaching grade S3 on the MRCS scale at the last follow-up.

**Table 3:**
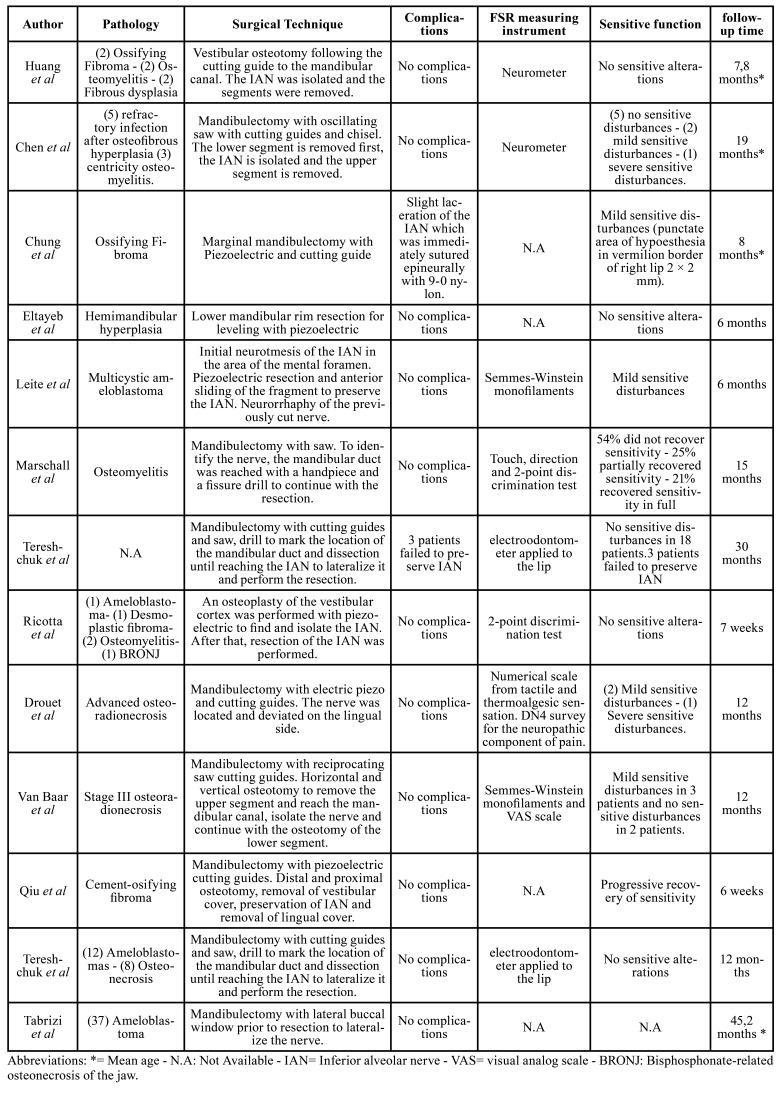
Description of surgical technique for preservation of the AIN assessment tool, treatment success and follow-up time.<

## Discussion

Despite the rapid evolution of mandibular reconstruction techniques after tumor resection, there has been little description of preservation of sensitivity in the lower lip. The post-surgical sequelae of mandibular resection are relevant because they severely affect the quality of life of patients (10). Preservation of the IAN in maxillofacial surgical procedures where surgical resection of the mandibular bone has been performed is an alternative that has shown successful outcomes in terms of a reduction in postoperative sequelae (4,10,13), and is currently positioned as a necessary and feasible procedure (10).

The surgical technique to preserve the IAN during mandibulectomy is simple and has allowed maintaining labiomental sensitivity during the postoperative period (13). In addition, it is easy to perform, has no residual effects in the donor area (in case of autologous transplantation), and has low additional economic costs (13).

In the clinical cases presented in this article, a piezoelectric mandibular splitting technique was performed, with which an anteroposterior cut was made in the superior and inferior area of the mandibular bone, achieving with the help of chisels separation of the mandibular vestibular cortex and allowing identification of the IAN. Subsequently, the nerve was carefully isolated and separated, and then the remaining segment of lingual cortex was resected. The aforementioned technique is similar to those used in the studies included in the review: Most of the authors described performing virtual planning prior to the surgery and using intraoperative cutting guides. These guides allow precise localization of the mandibular canal and the possibility of quantifying the necessary depth and distance of the cut, in addition to significantly shortening the intraoperative time and improving the results of the surgery (6,12). These cuts can be made with a saw, burs, or a piezoelectric system.

Chen *et al*. (10) described the use of virtual planning and a cutting guide. They fixed the guide on the mandibular inferior border to indicate the height of the osteotomy, which was performed with a reciprocating saw along the IAN until penetrating the cortex or to the previously determined depth. Subsequently, the inferior segment was removed with the help of chisels, exposing the IAN and carefully isolating it to continue with the osteotomy of the superior segment (10). Tabrizi *et al*. (8) preserve the IAN by making an osseous window in the buccal mandibular face to lateralize the IAN and to carry out the resection without the neurovascular bundle in between. On the other hand, Ricotta *et al*. (6) performed an osteoplasty of the vestibular cortex to isolate the IAN and then performed the mandibular resection. Leite *et al*. (4) cut the nerve with a scalpel at the level of the mental foramen, performing piezoelectric osteotomy of the anterior sector in front of it. The same procedure was performed posteriorly but preserving the nerve inside the canal, and then the resected fragment was slid anteriorly, keeping the inferior alveolar neurovascular bundle intact and performing a posterior neurorrhaphy with the distal segment of the mental nerve (4).

In addition to the technique used, it is important to determine the instrument with which the osteotomy will be performed. Several authors recommend that to ensure correct preservation of the IAN, it is safer to use a piezoelectric device, because it increases the precision of the osteotomy thanks to its strength and predicTable cutting speed compared with drills and saws that are very affected by the density of the bone tissue (12). Likewise, it has been reported that the use of this instrument causes less postoperative pain and inflammation and reduces the recovery time (12), does not damage soft tissue, and provides a clean surgical field with very little bleeding (14). Despite having a large number of advantages, piezoelectric systems have been heavily criticized because they require a longer intraoperative time (12). However, Landes *et al*. (16) carried out a study comparing this instrument with the conventional ones. They concluded that the surgical time was similar and piezoelectric systems caused less bleeding.

Regarding complications, only one laceration of the nerve was reported. It was immediately resolved with a 9-0 nylon epineural suture (12), which shows that these techniques are safe for the preservation of the IAN. In addition, Tereshchuk *et al*, reported that in 3 patients the nerve could not be preserved (2).

To check the success of nerve preservation, there are various methods for evaluating sensory function. For our cases, we used the MRCS: It was described and adapted by Mackinnon *et al*. (17) and is based on the degree of sensitivity and pain at different points of the affected area. We noted grade S4 and S3 sensitivity, which we considered successful for both patients. On this scale, grade S3 or higher is considered to be indicative of favorable sensory recovery (Table 2) (17). Zuniga *et al*. (18) described the neurosensory test (NST), which includes directional sensitivity to friction, two-point static discrimination, contact detection, pressure pain threshold, and pressure pain tolerance. On the other hand, several methods have been used to assess nerve sensitivity. Chen *et al*. (10) and Huang *et al*. (11) used a neurometer, which is an electrodiagnostic testing device with which the current perceptual threshold of the lower lip was measured. Leite *et al*. (4) and Van Baar *et al*. (5) used Semmes-Winstein filaments, and the latter group combined it with a visual analog scale. Finally, in their two studies, Tereshchuk *et al*. (2,7) applied an electro-odontometer to the lip, with a passive electrode attached to the patient's hand and an active electrode placed in the area innervated by the affected mental nerve. With this device, they recorded pre-surgical and post-surgical (up to 12 months) measurements.

Based on the included articles, there was a high success rate (Table 3). Most of the patients recovered sensitivity at a variable period of time post-surgery. However, we believe that the follow-up period should be longer than 6 months, because most IAN damage recovers within 12-24 weeks (19). In addition, Marschall *et al*. (9) reported that 100% of the patients included in their study had paresthesia of the IAN. After resective surgery and preservation of the neurovascular bundle, 21% recovered sensitivity completely, 25% recovered it partially, and 54% did not recover it within 6 months. Although a higher percentage did not achieve sensory recovery, it is noteworthy that 46% of the patients improved their initial condition, so presenting paresthesia prior to the procedure does not mean that IAN preservation surgery should not be performed.

Finally, it must be emphasized that this technique is not indicated for lesions that present some degree of perineural or nervous infiltration or a malignant character (4). However, Tereshchuk *et al*. (7) reported that this approach is also feasible for patients whose diagnosis involves a malignant tumor of the oral cavity, provided that strict criteria for the procedure are met: (a) The tumor must not have extended to the periodontal ligament or beyond the middle of the alveolar ridge. (b) The tumor must not have extended beyond the insertion point of the muscles of the floor of the mouth. (c) There must be no obvious invasion of the tumor into the mandible through the lingual cortical bone (2). On the other hand, an absolute contraindication for IAN preservation is invasion of malignant tumors within the mandible (7). Considering these criteria, Tereshchuk *et al*. (2) consider that the mandibular canal has its own cortical bone, which is an additional barrier to tumor spread. Nevertheless, the preservation of the IAN in patients with cancer is still a pending research topic (7).

Currently, it is imperative to perform maneuvers that allow locating and isolating the IAN when performing resective therapies of the mandible, in order to guarantee its indemnity and thus complete recovery in the operated patients, improving their quality of life considerably. In addition, nerve preservation is usually quick, is simple to perform, has a low associated economic cost, and has a low percentage of complications, most of which are described in the intraoperative stage and can be easily resolved. Moreover, recovery of postoperative sensitivity has been reported in patients who had no response to stimuli prior to resective treatment, when effective nerve preservation was performed. For successful preservation, authors have emphasized virtual preoperative planning and the elaboration of surgical guides that allow the surgery to be performed more precisely. The use of piezoelectric instruments has been described as the most beneficial way to make cuts in the mandibular cortices due to their numerous advantages over other instruments such as saws and cutting burs. Although there is a lack of studies supporting the advantages of IAN preservation in resective therapies, we conclude that it is a beneficial technique that should be considered by surgeons in cases where IAN indemnity is at risk.
